# Population Pharmacokinetic and Exposure‐Response Analysis of Vancomycin Nephrotoxicity in Cystic Fibrosis Patients

**DOI:** 10.1002/ppul.71748

**Published:** 2026-07-27

**Authors:** Jessica Barry, J. G. C. van Hasselt, Michael Evans, Elizabeth B. Hirsch, Jordan Dunitz, Sarah Shockley, Joanne Billings, Sílvia M. Illamola

**Affiliations:** ^1^ Department of Experimental and Clinical Pharmacology, College of Pharmacy University of Minnesota Minneapolis Minnesota USA; ^2^ Leiden University Leiden the Netherlands; ^3^ Clinical and Translational Science Institute University of Minnesota Minneapolis Minnesota USA; ^4^ The Minnesota Cystic Fibrosis Center, Department of Medicine University of Minnesota School of Medicine Minneapolis Minnesota USA; ^5^ Division of Pulmonary, Allergy, Critical Care and Sleep Medicine University of Minnesota Minneapolis Minnesota USA

**Keywords:** cystic fibrosis, nephrotoxicity, pharmacodynamics, population pharmacokinetics, vancomycin

## Abstract

**Introduction:**

Acute pulmonary exacerbations (APE) in persons with cystic fibrosis (PwCF) are associated with a reduction in long‐term pulmonary function. Vancomycin is recommended as first‐line treatment of methicillin‐resistant *Staphylococcus aureus* ‐APE in PwCF. While vancomycin poses a significant risk of nephrotoxicity, the relationship between vancomycin exposure and nephrotoxicity in PwCF has not been characterized.

**Aims:**

We aimed to establish the relationship between vancomycin exposure and onset of nephrotoxicity in PwCF, and to identify predictors of nephrotoxicity.

**Methods:**

This was a retrospective study among 52 adults PwCF. Nephrotoxicity events were defined based on the RIFLE criteria, as a serum creatinine increase of 1.5 times from baseline or a ≥ 25% decrease in glomerular filtration rate. A population pharmacokinetic (popPK) model was used to derive exposure measures (i.e., area‐under the concentration‐time curve (AUC)).

**Results:**

A total of 590 vancomycin serum concentrations were available for analysis. A one‐compartment model best describes vancomycin pharmacokinetics. Creatinine clearance was included as a significant covariate in the clearance. The popPK model allowed quantification of cumulative AUC (AUC_cum_) and AUC_24_ values, and AUC normalized (AUC_norm_) and cumulative dose were further calculated. Each variable of interest was used to fit a generalized linear mixed‐effects (GLM) model. The GLM model identified AUC_cum_ as a significant predictor of vancomycin nephrotoxicity (*p* < 0.01). Additionally, patients with more than one dosing occasion were significantly more likely to experience nephrotoxicity (*p* < 0.01). Based on the final AUC_cum_ model, the odds of experiencing a nephrotoxicity event increased by 32.6% (95% CI: 11.0%–58.4%) for each doubling of AUC_cum_.

**Discussion:**

Our results demonstrate an association between increased vancomycin exposures and risk of nephrotoxicity. This study provides a foundation for optimizing personalized vancomycin dosing strategies in PwCF to balance efficacy and nephrotoxicity risk.

**Abbreviations**
AKI: acute kidney injury
APE: acute pulmonary exacerbations
AUC: area‐under the concentration‐time curve
AUC24: AUC for 0–24 h
AUCcum: cumulative AUC
CF: cystic fibrosis
CL: clearance
CrCL: creatinine clearance
CWRES: conditional weighted residuals
D24/CL: dose‐24h/clearance
eGFR: estimated glomerular filtration rate
GFR: glomerular filtration rate
GOF: goodness of fit
IIV: Interindividual variability
IOV: Interoccasion variability
IPRED: individual predicted
LLOQ: lower limit of quantification
MIC: minimal inhibitory concentration
MRSA: Methicillin‐resistant Staphylococcus aureus
pc‐VPC: prediction‐corrected visual predictive check
PK: pharmacokinetic
PopPK: Population pharmacokinetics
PRED: population predicted
PwCF: persons with cystic fibrosis
RIFLE: risk‐injury‐failure‐loss‐end‐stage
RUV: residual unexplained variability
SCr: serum creatinine
TAD: time after dose
Vd: volume of distribution

## 1 Introduction

Persons with cystic fibrosis (PwCF) experience chronic airway infections with acute symptom aggravations called acute pulmonary exacerbations (APE), which reduce patients' quality of life and result in pulmonary function consequences, resulting in a decline in long‐term pulmonary function [1, 2, 3]. Methicillin‐resistant *Staphylococcus aureus* (MRSA) is often responsible for these infections [4]. MRSA is increasing in prevalence in cystic fibrosis (CF) infections, with a prevalence rate of 26.5% in PwCF in the United States [5, 6].

Antibiotic therapy for the treatment of APE is directed toward specific pathogens identified in culture‐based techniques [7]. Drug resistance and efficacy are of particular concern given the limited number of antibiotics available to treat MRSA. Although vancomycin is recommended as a first‐line treatment option for MRSA‐associated APE per national guidelines, limited data support its use in PwCF [8, 9, 10]. Furthermore, the 2009 Cystic Fibrosis Foundation Guidelines do not address the treatment of MRSA‐associated APE [7].

Vancomycin is associated with a significant risk of nephrotoxicity [11, 12, 13]. Animal studies suggest vancomycin accumulation as a mechanism of nephrotoxicity [14]. However, several studies have identified risk factors for the development of acute kidney injury (AKI) in patients treated with vancomycin [11, 15, 16, 17]. These risk factors, including higher vancomycin doses and trough concentrations, longer durations of treatment, and concomitant administration of other nephrotoxic drugs, are common in PwCF, who may receive repeated aminoglycoside and/or vancomycin treatment courses over their lifetime [18]. While the incidence of vancomycin‐associated AKI has been studied in non‐CF individuals, it has not been quantified in PwCF [11, 12, 13, 16, 19, 20]. The establishment of personalized vancomycin dosing strategies for PwCF needs to optimally balance sufficient efficacy while reducing the risk of nephrotoxicity.

The relationship between vancomycin exposure and nephrotoxicity has been investigated in non‐CF individuals [19, 20]. Cumulative AUC (AUC_cum_) can be used to quantify exposure over time, and AUC for 0–24 h (AUC_24_) is also often used as a target of vancomycin exposure [20]. Both markers have been investigated as predictors of aminoglycoside‐induced nephrotoxicity in non‐CF individuals [20, 21]. There is evidence that the risk of AKI increases as vancomycin AUC_24_ increases, especially when vancomycin AUC_24_ is greater than 700 mg h/L [19]. Therefore, a recent guideline for vancomycin treatment in non‐CF individuals recommends a target AUC_24_ to minimal inhibitory concentration (MIC) ratio (AUC_24_/MIC) of 400–600 mg*hour/L [10]. The relationship between vancomycin exposure and nephrotoxicity in PwCF has not been robustly characterized. Using a toxicity target non‐specific to PwCF may lead to an increased rate of nephrotoxicity among this population. Therefore, it is not currently possible to rationally optimize antibiotic dosing strategies in PwCF to minimize nephrotoxicity rates.

Population pharmacokinetics (popPK) and exposure‐response modeling are commonly used methods to quantify relationships between drug exposure and effects (e.g., nephrotoxicity) [16, 22]. Further identification of person‐specific factors that may explain this relationship will enable the prediction of vancomycin nephrotoxicity in PwCF, the first step towards the determination of individual optimal vancomycin doses to increase efficacy and decrease toxicity.

This study aimed to describe vancomycin nephrotoxicity prevalence in PwCF, to establish the relationship between measures of vancomycin exposure and the development of nephrotoxicity in PwCF, and to identify predictors of nephrotoxicity.

## 2 Methods

### 2.1 Study Population and Data Collection

Retrospective data were available from the Minnesota Cystic Fibrosis Center. Inclusion criteria required subjects: (1) had a confirmed CF diagnosis; (2) received intravenous vancomycin; (3) were ≥ 12 years old; (4) were hospitalized for an APE between January 2011 and June 2020; and (5) had MRSA‐positive culture within 30 days of hospitalization. Pregnant patients and those who used inhaled vancomycin were excluded. Patients could start vancomycin treatment either in an inpatient or outpatient setting, with possible setting changes during treatment. Data, including treatment regimen, vancomycin concentrations, demographics (e.g., age, sex, weight), concomitant medications, laboratory values (e.g., serum creatinine, liver enzymes), and MIC culture results, were collected from electronic health records (EHR). We identified concomitant medications, classified as nephrotoxic based on Naughton C et al. definition [23], administered within 3 days of a vancomycin blood concentration. Potential outliers were manually reviewed in the EHR. Estimated glomerular filtration rate (eGFR), as a measure of kidney function, was calculated from the patient's measured serum creatinine (SCr) and patient demographics using the CKD‐EPI equation [24]. Baseline SCr was the SCr at treatment start. This study (IRB number STUDY0011946) was reviewed by the University of Minnesota Institutional Review Board and met the category for exemption as secondary research for which consent was not required. Data were extracted by the Best Practices Integrated Informatics Core (BPIC) at the University of Minnesota.

### 2.2 Dosing and Sampling

Vancomycin was administered following a standardized protocol based on the patient's weight and creatinine clearance (CrCL). CrCL, a measure of the rate creatinine is cleared by the body, was calculated from the patient's measured SCr and patient's demographics using the Cockcroft‐Gault equation [25]. Vancomycin blood concentrations were monitored throughout the treatment using trough serum concentrations following the institution's protocol. Specifically, the first vancomycin blood concentration is generally drawn after reaching steady state (36–72 h), and then, vancomycin treatment is monitored by trough serum concentrations drawn within 30 min prior to the next vancomycin dose. AUC‐guided dosing was not used as the data in our study were collected (2011–2020) before the publication of the consensus guidelines recommending its use in 2020. Many subjects had multiple instances of dosing, defined as dosing occasions, describing vancomycin treatments separated by more than 14 days.

### 2.3 Bioanalysis

Samples were processed and quantified by the institutional CLIA‐approved clinical laboratory. Vancomycin concentrations were measured using a competitive immunoassay, with > 90% analyzed at a lower limit of quantification (LLOQ) of 0.8 mg/L, and < 10% at 5.0 mg/L. SCr concentrations were measured using an enzymatic assay, with > 90% at an LLOQ of 0.14 mg/dL, and < 10% at 0.15 mg/dL. The vancomycin MIC for the *Staphylococcus* species isolates was determined using a modified broth microdilution method via VITEK2 (BioMérieux, Durham, North Carolina). For any *Staphylococcus* isolate with a vancomycin MIC > 4 mg/L, confirmatory testing was performed using E‐test (BioMérieux, Durham, North Carolina) on Mueller‐Hinton agar.

### 2.4 Pharmacokinetic Model Development

The popPK analysis was performed using a non‐linear mixed effects modeling approach implemented in the software package NONMEM (version 7) (ICON Development Solutions), using the first‐order conditional estimation method with interaction. We used Perl‐speaks‐NONMEM (PsN) (version 3.5.2) and R (version 4.1.0) as supporting software packages for model building.

One and two‐compartment models were evaluated based on previous published results, and variability (interindividual (IIV) and interoccasion (IOV)) and different error models were tested [26, 27, 28]. IIV was assumed to be log‐normally distributed and assessed for pharmacokinetic (PK) model parameters using an exponential relationship. IOV was modeled similarly, under the assumption of log‐normality, and was tested for incorporation into the model as an exponential random effect on CL across all occasions. Additive, proportional, and combined models were considered for the quantification of residual unexplained variability (RUV).

Model development was guided by the decrease in the −2 log likelihood using a statistical significance criterion of a *P* value of < 0.01 (likelihood ratio test) and the precision of the parameter estimates. Goodness of fit (GOF) plots were used to assess structural model fit and model bias. During the popPK model‐building process, nested models were compared using their objective function and the likelihood ratio test, where the difference between the objective functions is assumed to be approximately chi‐square distributed with one degree of freedom. A decrease of 6.63 or more in the minimum value of the objective function was thus considered significant at 95% confidence.

Demographic and clinical covariates, including age, weight, height, race, sex, ethnicity, alanine transaminase, aspartate transferase, and creatinine clearance, were tested in the model to explain IIV on PK parameters. Time‐varying measures (e.g., weight) were updated over time in the clinical record. Covariate effects were introduced using stepwise forward addition followed by stepwise backward elimination procedures, with *p* < 0.01 and *p* < 0.001 as thresholds, respectively. Additionally, a covariate's effect on the decrease of the IIV was considered relevant for inclusion in the model, as well as physiological plausibility.

### 2.5 Model Evaluation

The choice of the structural model was guided by GOF diagnostics and the estimated precision of PK parameters. Additionally, models were evaluated using a prediction‐corrected visual predictive check (pc‐VPC) based on 1000 replicates of the dataset. The precision of the parameter estimates of the final popPK model was evaluated using a nonparametric bootstrap analysis (*n* = 2000) and the Perl‐speaks‐NONMEM (PsN) (version 3.5.2) program.

### 2.6 Exposure‐Response Modeling

Nephrotoxicity was defined using the Risk‐Injury‐Failure‐Loss‐End‐stage kidney disease (RIFLE) criteria, based on changes in SCr, urine output, and GFR. We used only changes in SCr and GFR to identify and categorize AKI into the first three severity levels: “Risk” (SCr increase 1.5 times baseline or a GFR decrease > 25%), “Injury” (SCr increase by 2 times baseline or a GFR decrease > 50%), and “Failure” (SCr increase by 3 times baseline or a GFR decrease > 75%) [29, 30]. No patients experienced the two most severe categories of RIFLE (“Loss” and “End‐stage Kidney Disease”). As the timing and frequency of SCr measurements varied between individuals, we standardized nephrotoxicity evaluation time points at 24‐h intervals during vancomycin treatment. If SCr values were missing at these time points, we imputed them using a linear interpolation method to appropriately manage the uneven time intervals associated with real‐world clinical data (R, zoo package). Nephrotoxicity events were determined by our model and not necessarily clinically documented.

Vancomycin exposure measures were captured using the developed popPK model. Occasion‐specific AUC_cum_ was calculated for the same 24‐h time points as for nephrotoxicity evaluation in NONMEM (Supporting information S1: Suppl. 1). For these 24‐h time points, we also calculated (1) AUC_24_ using (a) estimated individual PK parameters obtained from the developed popPK model by dose‐24h/clearance (CL) (D_24_/CL). AUC_24_ was a dynamic value updated with any changes to the dose during the occasion; and (b) AUC_24_ was the difference between AUC_cum_ at a specific time point and AUC_cum_ at the previous 24 h. (2) AUC_cum_ normalized by treatment duration (AUC_norm_) by dividing AUC_cum_ by the treatment duration at each time point. Finally, the cumulative dose for each occasion was calculated similarly to AUC_cum_, increasing every time a patient would receive their 24 h of dosing.

A mixed‐effects logistic regression modeled the relationship between vancomycin exposure (i.e., AUC_cum_, AUC_24_, AUC_norm_, and cumulative dose) and nephrotoxicity. Covariates that were not included in the PK model were tested at 90% confidence level (i.e., baseline GFR, weight, height, treatment duration, MIC, nephrotoxic medications, occasion, and time). Specifically, the occasion was evaluated as a continuous and as a categorical covariate. For the categorical analysis, we tested two binary groupings: (1) one occasion vs ≥ 2 occasions, and (2) one occasion, two occasions, or ≥ 3 occasions. Random effects, including patient and treatment occasion, were also tested for inclusion. The Hosmer‐Lemeshow Test was used to assess the observed and predicted event rate.

## 3 Results

### 3.1 Study Data

A total of 417 individuals had a confirmed CF diagnosis, received intravenous vancomycin, and were hospitalized for an APE between January 2011 and June 2020. From these 417 individuals, 52 individuals with 103 different dosing occasions met additional inclusion criteria of being ≥ 12 years old and having MRSA‐positive culture within 30 days of hospitalization, and were incorporated into the analysis (Table 1). Table 2 summarizes vancomycin treatment and concentrations for the whole population and stratified by inpatient/outpatient setting. Most individuals only received vancomycin inpatient (76.9%), while 19.2% of patients received vancomycin in both inpatient and outpatient settings (across 22 (21.4%) occasions). Most subjects received vancomycin on only one occasion (61%), and the number of dosing occasions for each individual ranged from 1 to 9. The median (range) treatment length of a dosing occasion was 10.7 (1.2–137.5) days. A total of 590 vancomycin concentrations were incorporated into the analysis. All included vancomycin and SCr concentrations were above their respective LLOQs. The median (range) of vancomycin sampling times was 8.9 (0.01–88) hours after the start of the infusion. The median (range) time between vancomycin occasions was 5 months (1 month–4 years). Specifically, 6 vancomycin concentrations were collected just after administration (0.01 h), 18 vancomycin concentrations in the first hour after the start of the infusion, and 40 vancomycin concentrations within 1–6 h after the start of the infusion. The median (range) of MIC values was 1.0 mg/L (0.5–2.0 mg/L). Patients included in this population received a number of concomitant medications, and 67.3% of individuals (48.6% of dosing occasions) received at least one dose of a nephrotoxic medication along with vancomycin. The most frequent nephrotoxic medications co‐administered were piperacillin/tazobactam (28% of occasions), diphenhydramine (27%), ceftazidime (16%), tacrolimus and tobramycin (12%), ranitidine (11%), and imipenem/cilastatin (11%). Sulfamethoxazole‐trimethoprim, an inhibitor of the tubular secretion of creatinine, was co‐administered along with vancomycin in 5 individuals (9.6%).

**Table 1:** Demographic and clinical characteristics of individuals included in the analysis.

Characteristics	Median (Range)	Count	Percent
Demographic characteristics			
Gender			
Male	—	27	52
Female	—	25	48
Age (years)	32.5 (15.8 − 73.8)	—	—
Race			
White	—	47	90
American Indian/Alaskan Native	—	1	2
Black	—	1	2
Asian	—	1	2
NA	—	2	4
Ethnicity			
Hispanic	—	1	2
Not Hispanic	—	51	98
Clinical characteristics			
Weight (kg)*	65 (39 − 102)	—	—
Height (cm)*	167.1 (147.3 – 189.2)	—	—
ALT	28.5 (13.8 – 500.8)	—	—
AST	24.1 (11.8 – 208.9)	—	—
CrCL	108.2 (18.8 – 251.3)	—	—
MIC	1 (0.5 – 2)	—	—

Abbreviations: ALT, alanine transferase; AST, aspartate transferase; CrCL, creatinine clearance; MIC, minimal inhibitory concentration.

*Weight and height measured at the start of treatment.

**Table 2:** Vancomycin treatment and concentration characteristics.

	All individuals	Only inpatient	Only outpatient
Number individuals (%)	52 (100)	40 (76.9)	2 (3.9)
Vancomycin treatment			
Number OCC (%)	103 (100)	79 (76.7)	2 (1.9)
Number OCC/individual*	1 (1–9)	1 (1–9)	2 (1–9)
Daily Dose (mg)#	2000 (500–6000)	2000 (500–6000)	3000 (1250–4500)
Frequency (hours)*		11.7 ± 7.44	8.26 ± 1.6
Vancomycin concentrations (mg/L)*	—	18.0 (2–53.8)	16.4 (2.1‐30)
Number concentrations (%)	590 (100)	457 (77.5)	133 (22.5)
Number conc./individual (%)	5 (1–76)	5 (1–44)	4 (1–52)

Abbreviations: OCC, occasion; SD, standard deviation.

*Expressed as median (range); # Expressed as mean (SD).

### 3.2 Model Development and Evaluation

A one‐compartment model with a combined error model best described the PK of vancomycin. The full NONMEM model files are provided as (Supporting information S1: Suppl. 1). Table 3 shows the final population PK parameter estimates. All fixed‐effect PK parameters were estimated with adequate precision, and high RUV was estimated (Table 3). IIV was included on CL and volume of distribution (V), while IOV was only on CL. IIV and IOV were described by an exponential error model. The condition number was 74.46, indicating that the model was not over‐parameterized. Shrinkage values for IIV on CL and IIV on V were 22.8% and 23.1%, respectively. Shrinkage of the residual model was < 10%. CrCL was found to be a significant covariate on CL, explaining 56% of the IIV in CL (Equation 1).

(1) $$CL_{i}=TVCL*{\left(\frac{CrCL_{i}}{CrCL_{\mathrm{median}}}\right)}^{\theta }$$

**Table 3:** Population pharmacokinetic parameter estimates for the final model.

Parameter	Final model		Bootstrap (*n* = 2000)		
	Estimate	%RSE	Estimate	CI 2.5%	CI 97.5%
CL (L/hr)	5.06	4.4	5.04	4.62	5.49
$\;\theta_{\mathrm{CrCL}}\sim \mathrm{CL}$	1.04	8.5	1.03	0.82	1.17
V (L)	92.7	12.4	91.6	71.6	116.9
Interindividual variability					
IIV CL (%CV)	26.9	27.9	26.3	8.5	39.5
IIV V (%CV)	67.1	13.9	65.1	42.6	86.7
Interoccasion variability					
IOV CL (%CV)	18.5	24.4	18.1	9.5	26.9
Residual unexplained variability					
Additive (mg/L)	2.6	23.6	2.6	0.86	3.5
Proportional (%)	18.7	21.6	18.6	8.7	25.4

Abbreviations: CI, confidence interval; CL, clearance; CrCL, creatinine clearance; IIV, interindividual variability; IOV, interoccasion variability; RSE, residual standard error; V, volume of distribution.

SCr measurements were taken with a mean of 2.97 ± 2.29 days between samples. Only 4.6% of the SCr data was imputed across more than 1 week.

**Figure 1:**
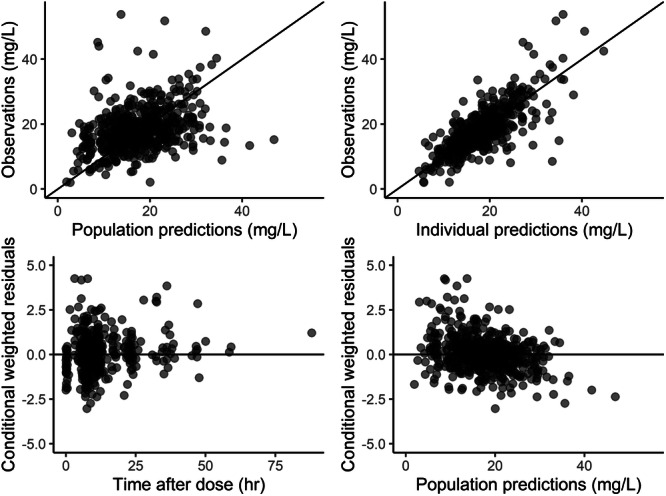
Goodness‐of‐fit plots of the final model. Population predictions (left top) and individual predictions (right top) versus observations and conditional weighted residuals (CWRES) versus time after dose (left bottom) and population predictions (left bottom).

**Figure 2:**
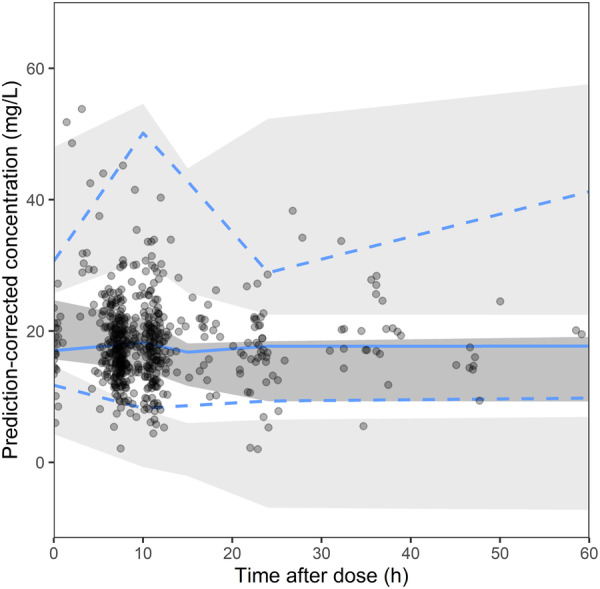
Prediction‐corrected visual predictive check (n = 1000) for the final population pharmacokinetic model. The dashed lines and gray areas represent the 2.5th and 97.5th percentiles of the observed and simulated concentration‐time profiles, respectively. The solid blue line and dark gray area represent the median observed and simulated concentration‐time profiles, respectively. [Color figure can be viewed at wileyonlinelibrary.com]

GOF plots of the final model showed good agreement between both the population (PRED) and individual (IPRED) predicted and observed concentrations, with most of the points around the identity line, thus not revealing any significant trends (Figure 1). A slight bias in the conditional weighted residuals (CWRES) vs PRED plot indicates potential for a better fit with a more complex structural model. The pc‐VPC captured the observed data adequately (Figure 2), and bootstrap analysis indicated that parameters could be estimated with adequate precision (Table 3).

### 3.3 Incidence of Nephrotoxicity

Sixteen individuals (31%) in 19 (18.4%) occasions experienced at least one nephrotoxic event throughout their vancomycin treatment (Supporting information S2: Suppl. 2). From these 16 individuals experiencing a nephrotoxic event, 62.5% had AUC_24_ exceeding the guideline‐recommended threshold (AUC_24_/MIC ≤ 400‐600 mg*hour/L). All 19 occasions met the “Risk” criteria; 9 met the “Injury” criteria, and 2 met the “Failure” criteria. Most of these individuals (81.2%) experienced only one nephrotoxic event, and the remaining individuals had two events each. Nephrotoxic events occurred at a median of 20 (range 2.5–63.5) days after the first dose of vancomycin treatment (first dosing occasion). However, 47% of nephrotoxic events occurred during a patient's second dosing occasion, at a median (range) of 10 (1–55) days after starting the second vancomycin dosing occasion.

**Figure 3:**
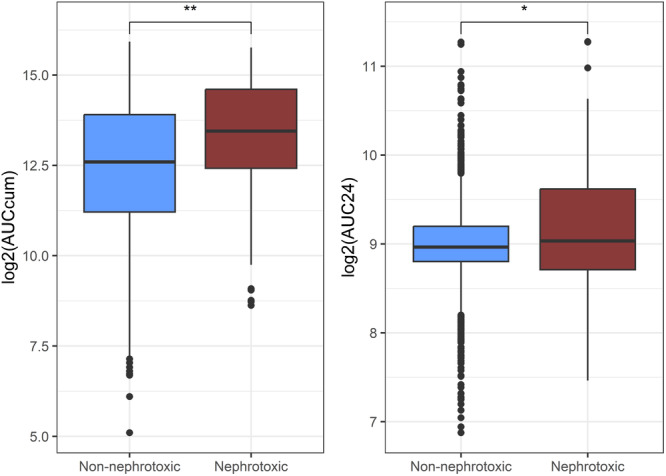
Boxplot of log2(AUC_cum_) (left) and log2(AUC_24_) (right) for non‐nephrotoxic and nephrotoxic occasions. *p < 0.05; **p < 0.001. [Color figure can be viewed at wileyonlinelibrary.com]

We compared vancomycin dose and exposure (AUC) between dosing occasions categorized as nephrotoxic and non‐nephrotoxic. None of these measures (i.e., AUC_24,_ AUC_cum_, AUC_norm_, vancomycin daily dose) was normally distributed, so we applied the non‐parametric test, the Mann‐Whitney *U*‐test, to test for differences. Vancomycin daily dose was significantly different between nephrotoxic (median [range] of 1700 mg [500–5100]) and non‐nephrotoxic dosing occasions (median [range] of 2250 mg [500–6000]) (*p* < 0.001). In 31.6% (6/19 occasions) of the cases, vancomycin dose was reduced after a model‐identified nephrotoxic event, and in 5.3% (1/19) of the cases, the treatment was stopped within 48 h. In 87.6% of dosing occasions, the vancomycin dose was changed during the first week of treatment (38.1% dose increase and 34.3% dose decrease). Duration of vancomycin treatment was statistically higher in the categorized nephrotoxic occasion group (median 18.3 days, range 4.4–138 days) compared to the non‐nephrotoxic group (median 9.2 days, range 1.2–93.3 days) (*p* < 0.001). Vancomycin outcome (either AUC_cum_ or AUC_24_) was statistically different between groups (*p* < 0.001) (Figure 3). From all the categorized nephrotoxic occasions, 31.6% of them met the risk threshold of AUC_24_ > 700 mg h/L as a marker of increased risk of AKI.

### 3.4 Exposure‐Response Analysis of Vancomycin‐Associated Nephrotoxicity

Nephrotoxicity was defined under the “Risk” criteria to ensure enough events to support quantification of a robust model [29]. After the model development, an estimated AUC_cum_, AUC_24_, AUC_norm_, and cumulative dose were quantified for every individual at each dosing occasion over time. The mixed‐effects logistic regression model showed that neither the AUC_24_ nor the cumulative dose models were statistically significant. The model, including AUC_norm_, found a negative correlation between AUC_norm_ and the risk of nephrotoxicity, and therefore, as these results are not physiologically plausible, the model was not retained (Supporting information S3: Suppl. 3). The logistic regression model with AUC_cum_ quantified a significant relationship between vancomycin exposure (i.e., AUC_cum_) and a reduction of eGFR (Table 4). Incorporating occasion as both a random and fixed effect provided a more significant p‐value and a reduction in random‐effect variance compared to the model incorporating occasion only as a random effect, despite the limited change in Akaike Information Criterion (AIC). The final model retained both patient and occasion as random effects, and occasion as a dichotomous covariate (one occasion vs ≥ two occasions), as a significant covariate. Based on the final AUC_cum_ model, for a patient receiving vancomycin for the first time, the odds of experiencing a nephrotoxicity event increased by 32.6% (95% CI: 11.0%–58.4%) for each doubling of AUC_cum_. For patients who were redosed with vancomycin on more than one occasion, their odds of experiencing a nephrotoxicity event are about 48 times higher (95% CI: 3–816 times). The binary Hosmer‐Lemeshow Test had a *p*‐value of 0.19, indicating there was no evidence that the observed and predicted frequencies of outcome were different. Overall, the model indicates the relevance and impact of recurring vancomycin treatments and cumulative exposure.

**Table 4:** Summary of the exposure‐response model cumulative AUC (AUC_cum_).

Parameter	Estimate (95% CI)	Std. Error	*p*‐value
Intercept	−11.9 (−15.6, −8.2)	1.89	3.5*10^−10^
Log2(AUC_cum_)	0.282 (0.104, 0.460)	0.091	0.002
OCC ≥ 2	3.89 (1.07, 6.71)	1.44	0.007

Group	Variance	Std. Dev.
Random effects: OCC:ID	6.27	2.5
Random effects: ID	22.7	4.8

*Note: AUC*_*cum*_: cumulative vancomycin area under the concentration‐time curve; OCC: visit occasion; ID: subject ID.

## 4 Conclusion

This study curated an extensive vancomycin data in PwCF, developing a one‐compartment popPK model of vancomycin in PwCF. The model identified CrCL as a significant predictor of CL. Most popPK vancomycin models in non‐CF individuals are one‐ or two‐compartment models, aligning with our findings [27]. A derived exposure‐response model showed a larger risk of nephrotoxicity with increased vancomycin exposure, aligning with prior studies in non‐CF individuals [20].

Vancomycin PK information in PwCF is scarce, with only one popPK study available [26, 31, 32, 33]. Our model's vancomycin CL estimate (5.06 L/h) is similar to the published popPK model (5.52 L/h). Both popPK models also share a comparable IIV estimate for CL (27% vs 23% in the Yellepeddi et al. model) [33]. We estimated a volume of distribution (V_d_) of 99.8 L/70 kg, significantly higher than the Vd estimated by Yellepeddi et al. (31.5 L/70 Kg), with a high IIV associated (67.1%), indicating the potential for high variability of this PK parameter in our population. High variability in vancomycin PK is not limited to PwCF; popPK models in adults show a large range of CL (0.33–8.75 L/h) and V_d_ (47.8–97.2 L) estimates across diseases [27]. However, as the institutional protocol suggests vancomycin treatment monitoring by trough serum concentrations, our dataset had a high percentage of trough concentrations, which can limit the accuracy of V_d_ estimation. To address this, we performed a sensitivity analysis by fixing V_d_ to a literature value [26] with a sufficient sampling to inform V_d_. This approach yielded similar PK parameter estimates to the final model (Supporting information S4: Suppl. 4). Therefore, we decided to estimate V_d_ instead of fixing it to a literature value. Significant covariates may have a different impact based on each population's characteristics. Our model found CrCL to impact CL values significantly in PwCF, while Yellepeddi et al. identified only weight [33]. CrCL is a common covariate identified as significant in vancomycin PK in non‐CF individuals, with other renal function markers, such as glomerular filtration rate, being less commonly used [27]. Age, body weight, and sex are also significant in some models in non‐CF individuals [27]. The potential for identification of additional covariates remains to further explain IIV on PK parameter estimates in PwCF.

We found a vancomycin nephrotoxicity incidence (31%) within the reported range (5% to 43%) [34]. Importantly, 81.2% of the individuals with vancomycin nephrotoxicity experienced it on just one dosing occasion, with approximately half of the nephrotoxic events occurring during the second dosing occasion, suggesting that having prior exposure to vancomycin may influence future nephrotoxicity risk. Additionally, approximately 60% of individuals had only one vancomycin dosing occasion, which limits the ability to observe the cumulative vancomycin nephrotoxic effect, as if a larger number of individuals with multiple occasions was available, the potential to see a larger number of nephrotoxic events after the first treatment occasion would likely have been increased.

Our exposure‐response analyses regarding nephrotoxicity provided key insight. We found a correlation between AUC_cum_ and the onset of nephrotoxicity. Additionally, the analysis confirmed the effect of prior exposure to vancomycin on future nephrotoxicity risk, as occasion was retained as a significant covariate in the model. Duration of treatment and vancomycin dose are highly correlated to AUC_cum_, confirming that both factors are already accounted for within the AUC_cum_ variable, and therefore confirming that they are both important factors contributing to nephrotoxicity. These findings aligned with similar work done in non‐CF populations, where vancomycin dose and AUC_cum_ have been linked to nephrotoxicity [12, 20, 35]. Of note, the negative correlation between AUC_norm_ and the onset of nephrotoxicity could potentially be caused by a bias in treatment duration due to the retrospective nature of the data. Although AUC_24_ is suggested to be used as a marker of vancomycin nephrotoxicity, we did not find a correlation between AUC_24_ and the onset of nephrotoxicity. However, 31.6% of nephrotoxic occasions that had an AUC_24_ greater than the suggested cut‐off (700 mg h/L) were classified as nephrotoxic, demonstrating that even if this predictive variable is used, the recommended cut‐off for non‐CF individuals may not be pertinent for PwCF. Indeed, PwCF may be at a higher risk of developing nephrotoxicity when treated with vancomycin, so a lower AUC_24_ cut‐off may be required. Importantly, AUC_cum_ was found to be the best marker for nephrotoxicity, and the popPK model described a low IIV in CL. Together, these findings may indicate that cumulative dose can be an easier and more practical predictor for nephrotoxicity in clinical practice. However, the use of AUC_cum_ instead of AUC_24_ as a vancomycin nephrotoxicity marker in clinical practice may limit the ability to early detect vancomycin‐associated nephrotoxicity and therefore the potential for a rapid dose adjustment to prevent AKI. Further efforts are needed to redefine the vancomycin toxicity target in PwCF.

Markers of renal function (i.e., SCr and CrCL) have been identified as significant predictors of nephrotoxicity [34, 36]. We did not identify any renal function marker as a predictor of nephrotoxicity in our regression model. We used SCr as a renal function marker and derived eGFR. However, SCr may lack the sensitivity to detect mild changes in renal function, which is a limitation for establishing a relationship between vancomycin exposure and the onset of nephrotoxicity based on SCr values. Other renal function markers (e.g., serum neutrophil gelatinase‐associated lipocalin, serum cystatin C, procalcitonin) that have not been validated in PwCF could serve as clinically significant toxicity biomarkers. Another limitation that complicates clinical interpretation of nephrotoxicity models is that studies may vary in the definition of nephrotoxicity. Different staging criteria based on eGFR or SCr changes may be used [21, 30]. These choices may influence estimates of nephrotoxicity incidence, resulting in varied understanding of what levels of vancomycin exposure are concerning.

Mechanism of changes in GFR may be caused by several reasons (e.g., interstitial nephritis, hydration status). However, due to the retrospective nature of this study, there is no reliable means to determine hydration status, nor was there a consistent protocol to confirm the mechanism of changes in the GFR, limiting the ability to identify other causes of nephrotoxicity besides vancomycin treatment. Previous studies in adults and children without CF identified the concomitant use of nephrotoxic medications as predictors of nephrotoxicity [12, 37, 38]. We did not find concomitant use of nephrotoxic agents as a predictor of nephrotoxicity among this cohort of PwCF. A potential reason could be that the medications classified as nephrotoxic vary within studies, and that prescribers limit their use when co‐administered with vancomycin.

Several factors may have contributed to the high proportional error from the residual unexplained variability found in our study. Although most of the individuals included in the study received inpatient‐only treatment, which is potentially associated with lower inaccuracy due to the automatic system for medication administration, retrospective data are variable in nature and may contain inaccuracies in dose and sampling information even when supplemented by manual review. When patients are hospitalized for repeated infections or for prolonged periods of time, several confounding mechanisms could also explain changes in SCr concentrations, such as lifestyle changes, nutrition, or modified treatment schedules. Additionally, due to the retrospective nature of expanded‐time data, assays and instruments used to measure vancomycin and SCr changed over time, adding to the inter‐assay error portion of the RUV. As our popPK model obtained significantly different estimates of V_d_ compared to the Yellepeddi et al. model, additional popPK studies in PwCF should be conducted [32]. The slight bias toward CWRES is potentially caused by abnormally high concentrations at long time‐after‐dose intervals. One potential cause is missing dosing records associated with the EHR used for the analyses, as these patients were all hospitalized at the time, and the accompanying manual reviews did not resolve this issue. An external dataset for future validation of the popPK model developed will also allow for its validation and adjustment if needed.

This work helps uncover the important relationship between vancomycin‐induced nephrotoxicity in PwCF and the predictors for nephrotoxicity. Further efforts are needed to redefine the vancomycin toxicity target in PwCF. Further studies can clarify the need for a CF‐specific model to predict and optimize vancomycin exposure in PwCF.

## Author Contributions

**Jessica Barry:** data curation, formal analysis, resources, software, writing – original draft, writing – review and editing. **J. G. C. van Hasselt:** conceptualization, methodology, writing – review and editing. **Michael Evans:** formal analysis, funding acquisition, writing – review and editing. **Elizabeth B. Hirsch:** conceptualization, writing – review and editing. **Jordan Dunitz:** conceptualization, resources, writing – review and editing. **Sarah Shockley:** writing – review and editing. **Joanne Billings:** writing – review and editing. **Sílvia M. Illamola:** conceptualization, data curation, formal analysis, funding acquisition, methodology, project administration, resources, software, supervision, writing – original draft, writing – review and editing.

## Ethics Statement

This study (IRB number STUDY0011946) was reviewed by the University of Minnesota Institutional Review Board and met the category for exemption as secondary research for which consent was not required.

## Conflicts of Interest

Jessica Barry, is a current employee of AbbVie, Inc. AbbVie was not involved in the production of this manuscript, and the majority of project work was completed during her affiliation with the University of Minnesota; this relationship is not related to the present work. Jordan Dunitz, is the Director of the Cystic Fibrosis Program at the University of Minnesota, APP Training Course Director, and received support from the Cystic Fibrosis Foundation for attending meetings and/or travel to the NACFC conference; none of these relationships are related to the present work. Elizabeth B. Hirsch received advisory board honoraria from GSK; this relationship is not related to the present work. Sílvia M Illamola received advisory board honoraria from NIH/NIDA as Safety Monitoring Committee member; this relationship is not related to the present work. Sarah Shockley reports a relationship with Vertex that includes consulting or advisory; this relationship is not related to the present work. The other authors declare no conflicts of interest.

## Supporting information


Supporting File 1

Supporting File 2

Supporting File 3

Supporting File 4

## Data Availability

The authors have nothing to report.
