# Supplementary material for: Population Pharmacokinetic and Exposure‐Response Analysis of Vancomycin Nephrotoxicity in Cystic Fibrosis Patients
Source: Pediatr Pulmonol. 2026 Jul 27;61(8):e71748. doi: 10.1002/ppul.71748 (PMC13406165; doi:10.1002/ppul.71748)
Supplement: Supplementary file 1 — Supporting File 1 [file PPUL-61-0-s001.docx]

**Supplemental Material 1 – Final NONMEM control streams**

;; 2. Description: 1-comp iv - combined [A+P] - IIV~CL+V

;; x1. Author: user

;; 1. Based on:

;; 3. Label: CF_VANCO

$PROBLEM 013comp - combined [A+P] - IIV~CL+V

;----------------------------------

$INPUT C ID DATE=DROP TIME AMT RATE DV II ADDL EVID MDV CMT OCC

XOCC AGE RACE SEX ETHN WT HT PDEV PDEV1 PDEV2 PDEV3 PDEV4

PDEV5 ALT AST UREA CREA CLCR BCRE BGFR DURA HOSP

;----------------------------------

$DATA data.csv IGNORE=@

;----------------------------------

$SUBROUTINE ADVAN6 TOL=6

$MODEL COMP=(CENTRAL) COMP=(DUMMY)

;----------------------------------

$PK

;---------define occassion---------

Q1 = 0

Q2 = 0

Q3 = 0

Q4 = 0

Q5 = 0

Q6 = 0

Q7 = 0

Q8 = 0

Q9 = 0

IF(OCC.EQ.1) Q1 = 1

IF(OCC.EQ.2) Q2 = 1

IF(OCC.EQ.3) Q3 = 1

IF(OCC.EQ.4) Q4 = 1

IF(OCC.EQ.5) Q5 = 1

IF(OCC.EQ.6) Q6 = 1

IF(OCC.EQ.7) Q7 = 1

IF(OCC.EQ.8) Q8 = 1

IF(OCC.EQ.9) Q9 = 1

;---------covariates---------------

;;; CLCLCR-DEFINITION START

CLCLCR = ((CLCR/114.13)**THETA(3))

;;; CLCLCR-DEFINITION END

;;; CL-RELATION START

CLCOV=CLCLCR

;;; CL-RELATION END

;----------------------------------

TVCL = THETA(1)

TVCL = TVCL*CLCOV

TVV = THETA(2)

IOVCL = Q1*ETA(3)+Q2*ETA(4)+Q3*ETA(5)+Q4*ETA(6)+Q5*ETA(7)+Q6*ETA(8)+Q7*ETA(9)+Q8*ETA(10)+Q9*ETA(11)

CL = TVCL * EXP(ETA(1)+IOVCL)

V = TVV * EXP(ETA(2))

AUC_CL = AMT/CL

S1 = V ;

IF(AMT.GT.0.AND.ADDL.EQ.0) THEN

TDOS=TIME

TAD=0

ENDIF

IF(ADDL.GT.0) THEN

TDOS=TIME + (ADDL*II)

TAD=0

ENDIF

IF(AMT.EQ.0) TAD=TIME-TDOS

;----------------------------------

$DES

DADT(1) = -CL*A(1)/V

DADT(2) = A(1)/V

;----------------------------------

$ERROR

IPRED = F

Y = IPRED * (1+ EPS(1)) + EPS(2)

IRES = DV-IPRED

A1 = A(1)

A2 = A(2)

AUC = A(2)

;----------------------------------

$THETA

(0,5.04094) ; CL

(0,95.1049) ; V

(-100,1.04051,100) ; CLCLCR1

;----------------------------------

$OMEGA

0.0694289 ; 1 CL ~30% IIV

0.365312 ; 2 V ~ 30% IIV

$OMEGA BLOCK(1)

0.033938 ; variance IOV CL

$OMEGA BLOCK(1) SAME

$OMEGA BLOCK(1) SAME

$OMEGA BLOCK(1) SAME

$OMEGA BLOCK(1) SAME

$OMEGA BLOCK(1) SAME

$OMEGA BLOCK(1) SAME

$OMEGA BLOCK(1) SAME

$OMEGA BLOCK(1) SAME

;----------------------------------

$SIGMA

0.0334186 ; [2-P]

7.04241 ; [1-A]

;----------------------------------

$ESTIMATION METHOD=1 INTERACTION MAXEVAL=9999 SIG=3 PRINT=5 NOABORT POSTHOC

$COVARIANCE PRINT=E UNCONDITIONAL

;----------------------------------

$TABLE ID TAD TIME DV AMT ADDL II MDV EVID IPRED CWRES AUC_CL AUC OCC

PDEV PDEV1 PDEV2 PDEV3 PDEV4 PDEV5 AGE RACE SEX ETHN WT HT

ALT AST UREA CREA CLCR HOSP BCRE BGFR DURA ONEHEADER

NOPRINT FILE=sdtab013_crcl_iovfi_auc_last.txt

$TABLE ID CL V ETA1 ETA2 ONEHEADER NOPRINT

FILE=patab013_crcl_iovfi_auc_last.txt

$TABLE ID OCC RACE SEX ETHN HOSP ONEHEADER NOPRINT FILE=catab013_crcl_iov_auc

$TABLE ID AGE WT HT ALT AST UREA CREA CLCR ONEHEADER NOPRINT

FILE=cotab013_crcl_iov_auc

;----------------------------------
