# Supplementary material for: Population Pharmacokinetic and Exposure‐Response Analysis of Vancomycin Nephrotoxicity in Cystic Fibrosis Patients
Source: Pediatr Pulmonol. 2026 Jul 27;61(8):e71748. doi: 10.1002/ppul.71748 (PMC13406165; doi:10.1002/ppul.71748)
Supplement: Supplementary file 2 — Supporting File 2 [file PPUL-61-0-s002.docx]

**Supplemental Material 2.** Individuals with nephrotoxicity events and associated information.

| **ID** | **OCC** | **Peak SCr Elevation (mg/dl) from baseline** | **Time after dose of peak elevation (hrs)** | **Vanc. AUC_24h_ prior to SCr elevation (mg*h/L)** | **log_2_AUC_cum_** | **Nephrotoxic comedications** |
| --- | --- | --- | --- | --- | --- | --- |
| 1 | 1 | 1.33 | 336 | 274.76 | 18.79 | - |
| 2 | 2 | 0.69 | 1320 | 905.14 | 19.53 | Ibuprofen |
| 3 | 1 | 1.72 | 1224 | 491.99 | 21.14 | Omeprazole |
| 4 | 2 | 1.52 | 72 | 1041.69 | 17.07 | - |
| 5 | 2 | 6.75 | 144 | 1378.70 | 17.5 | - |
| 5 | 3 | 5.42 | 312 | 993.65 | 18.75 | - |
| 6 | 2 | 1.94 | 552 | 486.55 | 18.62 | Sulfamethoxazole-Trimethoprim |
| 7 | 1 | 1.26 | 600 | 582.1 | 20.8 | - |
| 8 | 2 | 1.08 | 120 | 333.72 | 19.11 | Diclofenac |
| 8 | 3 | 1.25 | 1344 | 519.52 | 22.94 | - |
| 9 | 2 | 1.16 | 912 | 606.76 | 19.9 | - |
| 10 | 2 | 0.97 | 384 | 468.01 | 19.93 | - |
| 11 | 2 | 0.90 | 96 | 469.91 | 17.1 | - |
| 12 | 1 | 1.85 | 48 | 610.48 | 17.2 | - |
| 13 | 1 | 2.16 | 384 | 427.05 | 19.40 | Diphenhydramine |
| 14 | 1 | 2.47 | 96 | 1588.3 | 15.60 | Furosemide, Rantidine |
| 15 | 2 | 2.68 | 216 | 1131.1 | 18.58 | - |
| 16 | 3 | 2.96 | 72 | 717.4 | 17.04 | - |
| 16 | 4 | 5.63 | 168 | 856.5 | 18.16 | - |

*AUC_24_: area-under the concentration-time curve for 0-24 hours; AUC_cum_: cumulative area-under the concentration-time curve; ID: Individual; OCC: Occasion; SCr: serum creatinine;*
