# Supplementary material for: Population Pharmacokinetic and Exposure‐Response Analysis of Vancomycin Nephrotoxicity in Cystic Fibrosis Patients
Source: Pediatr Pulmonol. 2026 Jul 27;61(8):e71748. doi: 10.1002/ppul.71748 (PMC13406165; doi:10.1002/ppul.71748)
Supplement: Supplementary file 3 — Supporting File 3 [file PPUL-61-0-s004.docx]

**Supplemental Material 3.** Summary of the exposure-response models for different AUC calculations

| \| **Table I.** Summary of the exposure-response model of daily AUC (AUC_24_) calculated using daily dose/CL. \| \| \| \| \| --- \| --- \| --- \| --- \| \| **Parameter** \| **Estimate**  **(95% CI)** \| **Std. Error** \| **p-value** \| \| Intercept \| -5.51 (-9.61, -1.41) \| 2.09 \| 0.008 \| \| Log2(AUC_24_) \| -0.02 (-0.40, 0.37) \| 0.19 \| 0.932 \| \| **Group** \| **Variance** \| **Std. Dev.** \| \| \| Random effects: ID \| 15.16 \| 3.89 \|  \| \| *AUC_24_*: vancomycin daily area under the concentration-time curve, calculated using daily dose and clearance for each individual and occasion; OCC: visit occasion; ID: subject ID \| \| \| \|  \| **Table II.** Summary of the exposure-response model of daily AUC (AUC_24_) calculated as an AUC_cum_ subtraction. \| \| \| \| \| --- \| --- \| --- \| --- \| \| **Parameter** \| **Estimate**  **(95% CI)** \| **Std. Error** \| **p-value** \| \| Intercept \| -4.14 (-7.14, -1.14) \| 1.53 \| 0.007 \| \| Log2(AUC_24_) \| -0.20 (-0.54, 0.13) \| 0.17 \| 0.235 \| \| **Group** \| **Variance** \| **Std. Dev.** \| \| \| Random effects: ID \| 13.94 \| 3.73 \|  \| \| *AUC_24_*: vancomycin daily area under the concentration-time curve, calculated as AUC24 = (AUC(cum,t) – AUC(cum, t-1)) \| \| \| \| |
| --- | --- | --- | --- | --- | --- | --- | --- | --- | --- | --- | --- | --- | --- | --- | --- | --- | --- | --- | --- | --- | --- | --- | --- | --- | --- | --- | --- | --- | --- | --- | --- | --- | --- | --- | --- | --- | --- | --- | --- | --- | --- | --- | --- | --- | --- | --- | --- | --- | --- | --- | --- | --- | --- | --- | --- | --- |

| **Table III.** Summary of the exposure-response model of AUC normalized by time (AUC_norm_). | | | |
| --- | --- | --- | --- |
| **Parameter** | **Estimate**  **(95% CI)** | **Std. Error** | **p-value** |
| Intercept | -8.10 (-11.1, -5.08) | 1.54 | 1.5*10^-7^ |
| Log2(AUC_norm_) | -5.13 (-8.94, -1.32) | 1.94 | 0.01 |
| OCC ≥ 2 | 3.93 (1.14, 6.72) | 1.43 | 0.01 |
| **Group** | **Variance** | **Std. Dev.** | |
| Random effects: OCC:ID | 6.11 | 2.47 |  |
| Random effects: ID | 23.40 | 4.83 |  |
| *AUC_norm_*: normalized cumulative vancomycin area under the concentration-time curve, normalized by time; OCC: visit occasion; ID: subject ID | | | |

| **Table IV.** Summary of the exposure-response model cumulative dose. | | | |
| --- | --- | --- | --- |
| **Parameter** | **Estimate**  **(95% CI)** | **Std. Error** | **p-value** |
| Intercept | -7.56 (-11.1, -4.05) | 1.79 | 2.5*10^-5^ |
| Log(D_cumulative_) | 0.21 (-0.06, 0.48) | 0.14 | 0.13 |
| **Group** | **Variance** | **Std. Dev.** | |
| Random effects: ID | 14.8 | 3.84 |  |
| D_cumulative_: running cumulative vancomycin dose; OCC: visit occasion; ID: subject ID | | | |
