# Supplementary material for: Population Pharmacokinetic and Exposure‐Response Analysis of Vancomycin Nephrotoxicity in Cystic Fibrosis Patients
Source: Pediatr Pulmonol. 2026 Jul 27;61(8):e71748. doi: 10.1002/ppul.71748 (PMC13406165; doi:10.1002/ppul.71748)
Supplement: Supplementary file 4 — Supporting File 4 [file PPUL-61-0-s003.docx]

**Supplemental Material 4.** Sensitivity analysis for volume of distribution in the final model

|  | **Estimation V** | | **Fix V** | | | | |
| --- | --- | --- | --- | --- | --- | --- | --- |
| **Parameter** | **Estimate** | **%RSE** | **Estimate** | | | | **%RSE** |
| CL (L/hr) | 5.06 | 4.4 | 4.7 | | | | 4.5 |
| $\theta_{CrCL} \sim CL$ | 1.04 | 8.5 | 1.02 | | | | 8.3 |
| V (L) | 92.7 | 12.4 | 37.7 FIX | | | | - |
| **Interindividual variability** | | |  | | |  | |
| IIV CL (%CV) | 26.9 | 27.9 | 25.3 | | | | 27.2 |
| IIV V (%CV) | 67.1 | 13.9 | 89.0 | | | | 13.2 |
| **Interoccasion variability** | | |  | |  | | |
| IOV CL (%CV) | 18.5 | 24.4 | 17.6 | | | | 24.1 |
| **Residual unexplained variability** | | |  |  | | | |
| Additive (mg/L) | 2.6 | 23.6 | 2.6 | | | | 21.8 |
| Proportional (%) | 18.7 | 21.6 | 18.5 | | | | 21.8 |
| *CL clearance; CrCL creatinine clearance; V volume of distribution; IIV interindividual variability; IOV interoccasion variability; RSE residual standard error; CI confidence interval* | | | | | | | |
